# Prognostic significance of walking ability at hospital discharge for overall survival in newly diagnosed glioblastoma: a comparison across six peri-treatment assessments

**DOI:** 10.1007/s11060-026-05784-0

**Published:** 2026-09-11

**Authors:** Shuhei Tokiwa, Keisuke Natsume, Akira Yoshida, Hajime Yonezawa, Kentaro Kawamura, Kazuki Nakanishi, Nayuta Higa, Ryosuke Hanaya, Megumi Shimodozono

**Affiliations:** 1 Department of Rehabilitation, Kagoshima University Hospital, Kagoshima, Japan; 2 Department of Rehabilitation and Physical Medicine, Kagoshima University Graduate School of Medical and Dental Sciences, 8-35-1, Sakuragaoka, Kagoshima, 890-8520 Japan; 3 Department of Neurosurgery, Kagoshima University Graduate School of Medical and Dental Sciences, Kagoshima, Japan; 4 Department of Physical Therapy, School of Health Sciences, Faculty of Medicine, Kagoshima University, Kagoshima, Japan

**Keywords:** Functional ambulation category, Glioblastoma, Rehabilitation, Chemoradiotherapy, Prognosis

## Abstract

**Purpose:**

Walking ability is readily assessed at the bedside; however, whether it adds prognostic information beyond global performance status in glioblastoma (GBM) is unknown. We examined the Functional Ambulation Category (FAC) at six peri-treatment time points and whether discharge FAC is associated with overall survival (OS) independently of contemporaneous performance status.

**Methods:**

This retrospective single-center study included 185 adults with newly diagnosed GBM treated from 2011 to 2020. FAC was assessed preoperatively, on postoperative day 5, before chemoradiotherapy, at discharge, and 1 and 4 months after discharge. The primary analysis used a discharge landmark, with FAC grouped as 0–1, 2–4, and 5 and adjustment for age, sex, extent of resection, and discharge Karnofsky Performance Status (KPS). Incremental performance was assessed using Akaike information criterion (AIC) and Harrell’s C-index.

**Results:**

Higher FAC was associated with longer OS at all six time points, with the strongest univariable association at discharge. In the discharge-landmark model (*n* = 162; 138 deaths), adjusted hazard ratios versus FAC 0–1 were 0.45 (95% CI, 0.27–0.73) for FAC 2–4 and 0.33 (95% CI, 0.17–0.63) for FAC 5. Adding FAC improved fit and discrimination (ΔAIC, − 8.4; ΔC-index, + 0.022; *p* = 0.002). Estimates were unchanged after additional adjustment for bevacizumab and post-discharge temozolomide.

**Conclusion:**

Discharge FAC carries prognostic information not captured by contemporaneous global performance status and is a practical bedside marker at the transition to post-discharge care, rather than a stand-alone prediction tool.

**Registration:**

This study was not prospectively registered.

**Protocol:**

No prospective study protocol was prepared.

**Supplementary Information:**

The online version contains supplementary material available at https://doi.org/10.1007/s11060-026-05784-0.

## Introduction

Glioblastoma (GBM) is the most common and aggressive primary malignant brain tumor in adults [1, 2]. Despite standard multimodal therapy consisting of maximal safe resection followed by radiotherapy with concomitant and adjuvant temozolomide (the Stupp regimen), prognosis remains poor, with a median overall survival (OS) of approximately 12–15 months [1–3]. Clinical factors associated with survival therefore remain important. Several clinical prediction models have incorporated routinely available prognostic factors to stratify patients with newly diagnosed GBM [4]. Molecular markers such as O6-methylguanine-DNA methyltransferase (MGMT) promoter methylation are well-established prognostic and predictive factors [5, 6] but are not consistently available in retrospective cohorts, sustaining the importance of functional markers. Functional status is particularly relevant because it reflects a patient’s capacity to tolerate and complete intensive treatment [6, 7].

Performance status and physical activity have been associated with survival in GBM or malignant glioma [6–8], while rehabilitation- and discharge-related functional outcomes have been associated with survival and other clinical outcomes in brain tumor populations [9–12]. At our institution, patients with newly diagnosed GBM are managed through an integrated care pathway incorporating early postoperative rehabilitation and continued inpatient rehabilitation during chemoradiotherapy (CRT). Previous studies from our institution found this approach to be feasible and showed that better functional status at discharge was associated with longer OS, irrespective of age [11, 12]. This supports use of a simple measure of clinically meaningful changes in walking ability during routine care, particularly in brain tumor rehabilitation, where individualized multidisciplinary approaches are recommended [13, 14].

The Functional Ambulation Category (FAC) is well suited to this purpose. It is a six-point ordinal scale that classifies walking ability according to the level of physical assistance required and has demonstrated good reliability and responsiveness in neurological populations [15, 16]. In patients with brain tumors, the FAC has been used to assess walking independence at hospital discharge [17]. Because severe motor impairment, reduced consciousness, aphasia, and cognitive dysfunction often limit the feasibility of stopwatch-based walking tests in patients with GBM, the FAC may provide a more inclusive bedside assessment of walking ability [15–18]. Although several mobility-related measures, including balance and functional status measures, have been reported to be associated with outcomes in patients with GBM [18, 19], previous studies have not identified a significant association between walking-specific measures and prognosis [8, 18].

Another important consideration is the timing of functional assessment. Through the GBM treatment trajectory—from the preoperative period through early postoperative recovery, CRT initiation, hospital discharge, and post-discharge follow-up—walking ability may change substantially. In our previous study, activities of daily living (ADL) also changed markedly during the early treatment course, declining after surgery and subsequently recovering by discharge [11]. However, it remains unclear whether walking ability is associated with OS in patients with newly diagnosed GBM and, if so, which clinical time point provides the most informative prognostic value.

Therefore, we aimed to determine whether the FAC is associated with OS in newly diagnosed GBM and which of six peri-treatment assessments provides the strongest prognostic information. We hypothesized that discharge FAC would provide the strongest prognostic information among in-hospital assessments and examined its incremental value beyond contemporaneous discharge KPS.

## Methods

### Patient selection process

In this retrospective cohort study, we screened 225 consecutive adult admissions with suspected or newly diagnosed GBM at a single university hospital between January 2011 and December 2020. Histopathological diagnoses were based on the diagnostic criteria in use at the time of diagnosis. Systematic retrospective reclassification of the entire cohort according to the 2021 World Health Organization (WHO) Classification of Tumors of the Central Nervous System was not performed [20]. At our institution, isocitrate dehydrogenase (IDH) mutation status was assessed by immunohistochemistry in nearly all cases from 2016 onward; from 2018 onward, gene panel testing was also used in addition to immunohistochemistry. However, these molecular data were not systematically extracted or reconstructed across the entire historical cohort. Patients without available molecular information were retained on the basis of the histopathological diagnosis made at the time, without imputation or exclusion based on molecular status. MGMT promoter methylation testing was not routinely performed as part of clinical care and was rarely documented in clinical records. Eligible patients underwent surgery or biopsy, initiated standard concomitant CRT with temozolomide plus radiotherapy, and received continuous inpatient rehabilitation during the index hospitalization; CRT interruption or discontinuation was not an exclusion criterion. Forty cases outside this predefined index-care pathway were excluded, leaving 185 patients in the final analytic cohort; detailed exclusion reasons are provided in Online Resource 1. Of these, 110 received standard CRT and 75 received standard CRT plus bevacizumab. The final analytic cohort comprised 185 patients, including 110 and 75 treated with standard CRT and standard CRT plus bevacizumab, respectively.

### Treatment course and inpatient rehabilitation

Maximal safe resection or biopsy was performed shortly after admission. Extent of resection (EOR) was classified as biopsy, partial resection, subtotal resection (STR), or gross total resection (GTR). Biopsy was defined as diagnostic tissue sampling only; partial resection as < 80% resection; STR as ≥ 80% resection with residual enhancing tumor; and GTR as no residual enhancing tumor on early postoperative contrast-enhanced magnetic resonance imaging (MRI). EOR was determined primarily by comparing preoperative and early postoperative contrast-enhanced MRI, mainly contrast-enhanced T1-weighted images, according to the amount of residual enhancing tumor. Intraoperative visual estimates documented in the operative record were not used as the primary basis for EOR classification. Concomitant CRT was performed following institutional practice, consisting of temozolomide (75 mg/m² daily) with fractionated radiotherapy, with adjuvant temozolomide (150–200 mg/m² on days 1–5 of each 28-day cycle) administered thereafter as tolerated. Before July 2018, radiotherapy was generally delivered as 60 Gy in 30 fractions. From July 2018 onward, patients aged > 75 years generally received 40.5 Gy in 15 fractions, whereas 60 Gy in 30 fractions continued to be used for other patients. Bevacizumab was added at the discretion of the treating neurosurgeon.

Inpatient rehabilitation was initiated as early as medically feasible postoperatively, typically within 3 days, and continued throughout the index hospitalization. Programs were individualized according to neurological deficits, medical condition, fatigue, and treatment tolerance and included progressive mobilization, balance, transfer, gait, and ADL training [11, 12, 21].

### Outcome measures

The outcome was OS. For the six-time-point screening analyses, survival was calculated from surgery to death or last follow-up. Because discharge FAC was assessed later in the clinical course, the primary adjusted analysis used hospital discharge as a landmark, with survival calculated from discharge to death or last follow-up. Survival status was ascertained through August 31, 2025. FAC is a six-point ordinal scale (0–5) based on the degree of human assistance required for walking, from nonfunctional ambulation to independent walking on all surfaces [15]; full level definitions are provided in Online Resource 1. The FAC was assessed preoperatively, on postoperative day 5 (± 1), before CRT, at discharge, and 1 and 4 months after discharge. For the main dataset, the FAC was retrospectively assigned by one physical therapist from rehabilitation records, physicians’ notes, and nursing documentation of daily-life mobility.

To evaluate reproducibility, independently generated discharge FAC ratings from a second physical therapist were available for 71 patients from an unrelated retrospective investigation. Agreement was assessed using linear weighted Cohen’s kappa as the primary statistic and ICC(2,1) as a supplementary metric; full methods are provided in Online Resource 1.

### Statistical analyses

Associations between the FAC and OS were assessed using Kaplan–Meier curves, log-rank tests, and Cox proportional hazards models. For screening across the six assessment time points, the FAC was entered as an ordinal score, with hazard ratios estimated per 1-point increase. For the primary discharge analysis, the FAC was grouped as 0–1, 2–4, and 5 after assessment of its functional form; the full comparison of candidate representations is provided in Online Resource 1. Pairwise comparisons between discharge FAC groups used log-rank tests with Bonferroni correction.

Separate univariable Cox models were fitted for all six FAC time points using survival from surgery. The primary multivariable analysis used a discharge-landmark Cox model with FAC 0–1 as the reference and adjustment for age, sex, EOR, and discharge KPS (per 10-point increase). Incremental information beyond contemporaneous performance status was assessed by comparing the clinical model with and without discharge FAC using AIC, Harrell’s C-index, and a likelihood-ratio test [22, 23].

The four in-hospital FAC assessments were compared on a common basis by adding each three-group FAC measure separately to a fixed clinical reference model including age, sex, preoperative KPS, and EOR in a common complete-case subset. Systematic landmark sensitivity analyses aligned the survival origin with each assessment; full specifications are provided in Online Resource 1. Post-discharge FAC assessments were exploratory. Secondary subgroup analyses examined age, sex, preoperative KPS, and EOR, with interactions tested using product terms.

Follow-up duration was summarized using the reverse Kaplan–Meier method. Missing data were handled by complete-case analysis without imputation. The proportional hazards assumption was assessed using scaled Schoenfeld residuals; when non-proportionality was detected for a covariate, a sensitivity Cox model stratified by that covariate was fitted. Sensitivity analyses addressing bevacizumab, standard CRT without upfront bevacizumab, EOR coding, and post-discharge adjuvant temozolomide are detailed in Online Resource 1. Because exact adjuvant temozolomide initiation dates were unavailable, that covariate was used only in sensitivity analysis and not interpreted as a treatment-effect estimate. IDH status and MGMT promoter methylation were not modeled because complete molecular data were unavailable for a substantial proportion of this historical cohort and availability depended strongly on treatment era. Corticosteroid exposure was not modeled because dose, timing, duration, and tapering could not be reconstructed consistently across FAC assessments. Descriptive analyses used IBM SPSS Statistics (version 26; IBM Corp., Armonk, NY), and survival/model-comparison analyses were reproduced and extended in R. Reporting followed the Strengthening the Reporting of Observational Studies in Epidemiology Statement [24] (Online Resource 2).

## Results

### Baseline characteristics and survival differences according to discharge FAC groups

Baseline characteristics differed across discharge FAC groups (Table 1). Discharge KPS was available for 167 patients. Post-discharge adjuvant temozolomide was initiated in 24 of 40 patients (60.0%) with FAC 0–1, 62 of 69 (89.9%) with FAC 2–4, and 57 of 58 (98.3%) with FAC 5.

**Fig. 1:**
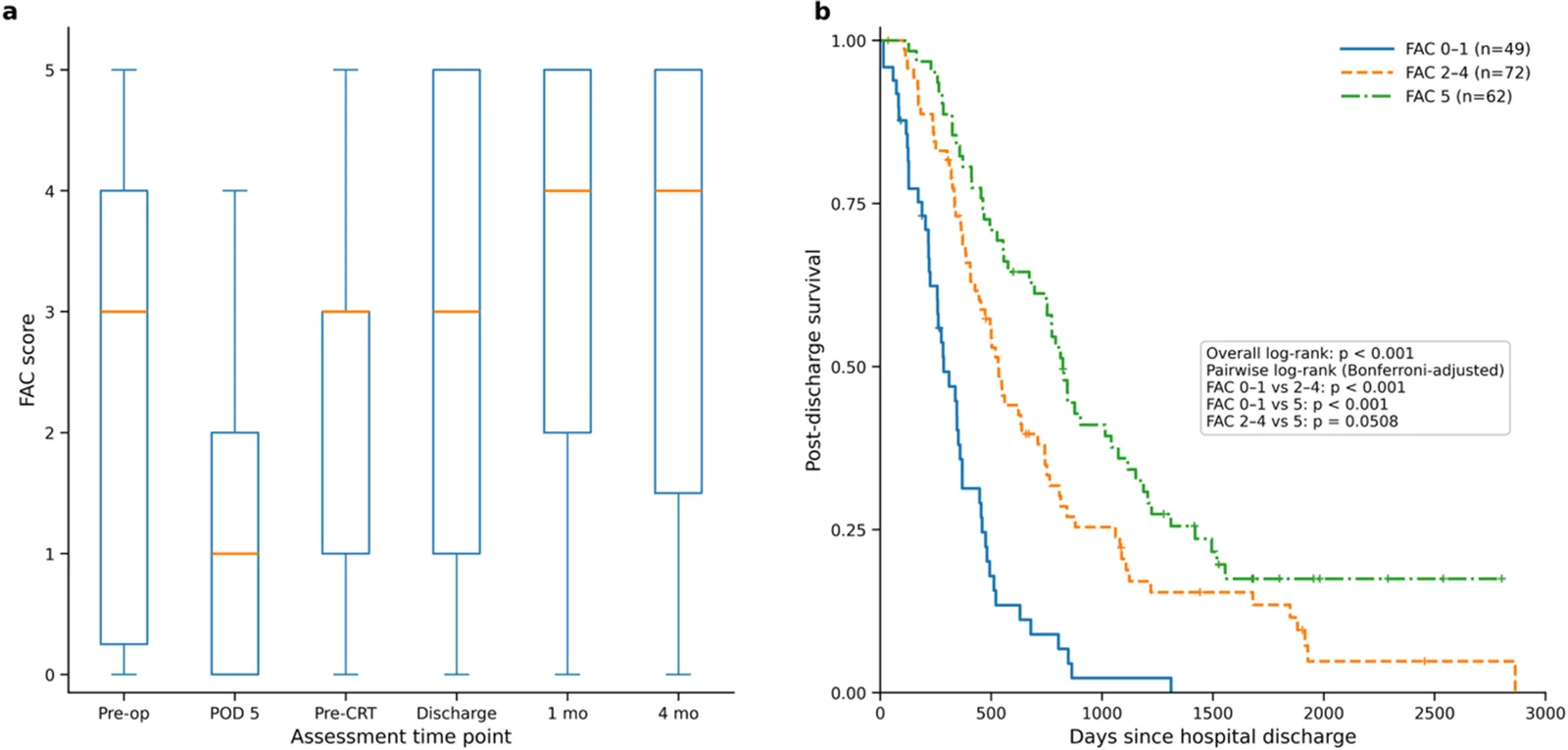
Trajectory of walking ability and discharge-landmark survival. (**a**) Box plots of Functional Ambulation Category (FAC) scores at six peri-treatment time points. Boxes indicate medians and interquartile ranges; whiskers indicate ranges. Numbers of evaluable patients were 182, 185, 185, 184, 141, and 123, respectively; each time point was summarized using available cases. Higher scores indicate greater walking independence. (**b**) Kaplan–Meier curves using hospital discharge as the landmark according to discharge FAC (0–1, 2–4, and 5). Tick marks indicate censored observations; survival time is shown from hospital discharge. The discharge-landmark analysis included data from 183 patients; 1 patient with available discharge FAC was excluded because a valid post-discharge survival interval could not be established from the recorded dates

Walking ability across the six assessment time points is shown in Fig. 1a. The single missing discharge FAC value reflected missing assessment data. Median follow-up by reverse Kaplan–Meier was 2,019 days. Median hospital stay was 63 (IQR, 53–71; range, 31–131) days, with 58 and 43 days from surgery and CRT initiation to discharge, respectively.

From surgery, the median OS was 346, 590, and 879 days for FAC 0–1, 2–4, and 5, respectively (overall log-rank *p* < 0.001). Using hospital discharge as the landmark, median post-discharge survival was 286 (95% CI, 257–369), 531 (95% CI, 445–741), and 823 (95% CI, 748–1,116) days, respectively (overall log-rank *p* < 0.001; Fig. 1b). Bonferroni-corrected pairwise comparisons showed differences for FAC 0–1 versus 2–4 and FAC 0–1 versus 5 (both *p* < 0.001), whereas the FAC 2–4 versus 5 comparison did not reach the corrected significance threshold (*p* = 0.051).

**Table 1:** Clinical characteristics according to discharge FAC group

Characteristic	Overall (*n* = 185)	FAC 0–1 (*n* = 50)	FAC 2–4 (*n* = 72)	FAC 5 (*n* = 62)
Age, mean (SD), y	67.1 (13.2)	72.7 (10.0)	70.1 (12.0)	59.1 (13.2)
Sex, n (%)				
Female	84 (45.4)	27 (54.0)	27 (37.5)	29 (46.8)
Male	101 (54.6)	23 (46.0)	45 (62.5)	33 (53.2)
Preoperative KPS, median (IQR), *n* = 152	60 (50–70)	50 (40–60)	60 (50–70)	70 (60–90)
Discharge KPS, median (IQR), *n* = 167	60 (50–80)	40 (30–40)	60 (60–70)	90 (60–90)
Tumor location, n (%), *n* = 183				
Frontal	47 (25.7)	10 (20.8)	19 (26.4)	17 (27.4)
Temporal	47 (25.7)	7 (14.6)	14 (19.4)	26 (41.9)
Parietal	22 (12.0)	5 (10.4)	7 (9.7)	10 (16.1)
Occipital	6 (3.3)	2 (4.2)	4 (5.6)	0 (0.0)
Deep/central	36 (19.7)	15 (31.2)	16 (22.2)	5 (8.1)
Multilobar	25 (13.7)	9 (18.8)	12 (16.7)	4 (6.5)
Hemisphere, n (%)				
Right	78 (42.2)	22 (44.0)	31 (43.1)	24 (38.7)
Left	82 (44.3)	16 (32.0)	33 (45.8)	33 (53.2)
Bilateral	22 (11.9)	11 (22.0)	6 (8.3)	5 (8.1)
Deep	3 (1.6)	1 (2.0)	2 (2.8)	0 (0.0)
EOR, n (%), *n* = 182				
Biopsy	29 (15.9)	14 (28.6)	10 (14.1)	5 (8.2)
Partial resection	55 (30.2)	21 (42.9)	22 (31.0)	12 (19.7)
Subtotal resection	45 (24.7)	6 (12.2)	19 (26.8)	19 (31.1)
Gross total resection	53 (29.1)	8 (16.3)	20 (28.2)	25 (41.0)
Treatment regimen, n (%)				
Standard CRT (TMZ)	110 (59.5)	26 (52.0)	48 (66.7)	35 (56.5)
Standard CRT+bevacizumab	75 (40.5)	24 (48.0)	24 (33.3)	27 (43.5)
Hospital stay, median (IQR); range, d, *n* = 182	63 (53–71); 31–131	70.5 (54.0–76.8); 39–131	60 (50–68); 37–104	63 (54.2–68.0); 31–107
Post-discharge adjuvant TMZ, n (%)^c^				
Yes	144 (85.7)	24 (60.0)	62 (89.9)	57 (98.3)
No	24 (14.3)	16 (40.0)	7 (10.1)	1 (1.7)

^a^Values are shown as mean (standard deviation), median (interquartile range), or n (%), as appropriate

^b^Percentages were calculated using available data for each variable. One patient with missing discharge FAC was excluded from group comparisons

^c^Post-discharge adjuvant TMZ data were available for 168 patients overall and for 167 patients with known discharge FAC; percentages for FAC groups were calculated using available data within each group

CRT, chemoradiotherapy; EOR, extent of resection; FAC, Functional Ambulation Category; IQR, interquartile range; KPS, Karnofsky Performance Status; TMZ, temozolomide; SD, standard deviation

Separate univariable Cox models using survival from surgery showed significant associations between higher FAC and longer OS at all six assessment time points, with the strongest association at discharge (HR per 1-point increase, 0.760; 95% CI, 0.697–0.830; *p* < 0.001; Table 2). Functional-form assessment supported use of the clinically interpretable three-group representation in the primary discharge model; full comparison results are provided in Online Resource 1.

**Table 2:** Univariable Cox regression analyses of FAC at six assessment time points for overall survival

FAC time point^a^	Cases, *n*	Events, *n*	HR	95% CI	Wald χ²	*p* value
Preoperative	182	158	0.858	0.786–0.936	11.814	0.001
Postoperative day 5	185	160	0.803	0.710–0.909	12.155	< 0.001
Before CRT	185	160	0.796	0.723–0.877	21.477	< 0.001
At discharge	184	159	0.761	0.697–0.830	37.464	< 0.001
1 month after discharge	141	119	0.766	0.693–0.847	26.907	< 0.001
4 months after discharge	123	104	0.800	0.722–0.886	18.236	< 0.001

^a^FAC was entered as an ordinal variable (0–5), and hazard ratios are shown per 1-point increase. Cases with missing FAC at a given time point were excluded from the corresponding analysis

CI, confidence interval; FAC, Functional Ambulation Category; HR, hazard ratio

### Primary discharge-landmark survival analysis

In the primary discharge-landmark Cox model (*n* = 162; 138 deaths), discharge FAC remained associated with survival after adjustment for age, sex, EOR, and contemporaneous discharge KPS (Table 3A). Compared with FAC 0–1, the adjusted HR was 0.445 (95% CI, 0.271–0.730; *p* = 0.001) for FAC 2–4 and 0.327 (95% CI, 0.169–0.632; *p* < 0.001) for FAC 5. Discharge KPS was also independently associated with survival (HR per 10-point increase, 0.859; 95% CI, 0.758–0.972; *p* = 0.016). Male sex was associated with higher mortality, whereas greater EOR was associated with lower mortality (Table 3A). Adding discharge FAC to the clinical model improved fit and discrimination (ΔAIC, − 8.4; ΔC-index, + 0.022; likelihood-ratio *p* = 0.002; Table 3B).

In the common complete-case comparison of the four in-hospital assessments (*n* = 146; 128 deaths), discharge FAC produced the largest improvement over the reference clinical model (ΔAIC, − 20.1; ΔC-index, + 0.043; likelihood-ratio *p* < 0.001), whereas improvements were smaller at earlier assessments (Table 3C). Systematic landmark analyses showed the same temporal pattern, with the strongest FAC contribution at discharge (Online Resource 1).

The primary FAC estimates were stable across sensitivity analyses, including adjustment for bevacizumab, restriction to standard CRT without upfront bevacizumab, adjustment for post-discharge adjuvant temozolomide, and categorical EOR modeling (Online Resource 1). The proportional hazards assumption held for discharge FAC (*p* = 0.129) but not for EOR (*p* = 0.006). When EOR was entered as a stratification factor, rather than as a covariate, the FAC estimates were unchanged (FAC 2–4 vs. 0–1: adjusted HR, 0.487; 95% CI, 0.289–0.820; FAC 5 vs. 0–1: adjusted HR, 0.364; 95% CI, 0.181–0.730).

**Table 3:** Primary discharge-landmark model and incremental model performance

A. Primary discharge-landmark Cox regression model^a^				
Predictor	Adjusted HR	95% CI	p value	
Discharge FAC: 0–1	Reference			
Discharge FAC: 2–4	0.445	0.271–0.730	0.001	
Discharge FAC: 5	0.327	0.169–0.632	< 0.001	
Age, per 1 year	0.994	0.979–1.009	0.430	
Male sex	1.471	1.032–2.096	0.033	
EOR, per category	0.741	0.621–0.886	0.001	
Discharge KPS, per 10 points	0.859	0.758–0.972	0.016	

B. Incremental prognostic value of discharge FAC beyond discharge KPS^b^				
Model	AIC	ΔAIC	C-index	ΔC-index
Age + sex + EOR + discharge KPS	1134.8	Reference	0.692	Reference
+ discharge FAC (0–1/2–4/5)	1126.4	−8.4	0.715	+ 0.022

C. Incremental prognostic value of each in-hospital FAC assessment^c^				
Model	AIC	ΔAIC	C-index	ΔC-index
Reference clinical model	1046.9	Reference	0.641	Reference
+ Preoperative FAC	1050.5	+ 3.5	0.645	+ 0.003
+ Postoperative day 5 FAC	1045.5	−1.4	0.641	0.000
+ Before CRT FAC	1044.9	−2.0	0.652	+ 0.010
+ Discharge FAC	1026.8	−20.1	0.684	+ 0.043

^a^The primary model used survival from hospital discharge and complete cases for discharge FAC, age, sex, EOR, and discharge KPS (n=162; deaths=138). FAC 0–1 was the reference category. EOR was coded ordinally from biopsy to gross total resection, and discharge KPS was entered per 10-point increase

^b^Both models used the same 162 complete cases. The likelihood-ratio test for adding discharge FAC was p=0.002. Lower AIC and higher C-index indicate better model performance

^c^The in-hospital comparison used a common complete-case subset (n=146; deaths=128) and a reference model including age, sex, preoperative KPS, and EOR. FAC at each time point was entered using the 0–1 / 2–4 / 5 classification. Likelihood-ratio p values versus the reference model were 0.783, 0.064, 0.049, and <0.001 for preoperative, postoperative day 5, pre-CRT, and discharge FAC, respectively

AIC, Akaike information criterion; CI, confidence interval; EOR, extent of resection; FAC, Functional Ambulation Category; HR, hazard ratio; KPS, Karnofsky Performance Status

### Subgroup analyses

**Fig. 2:**
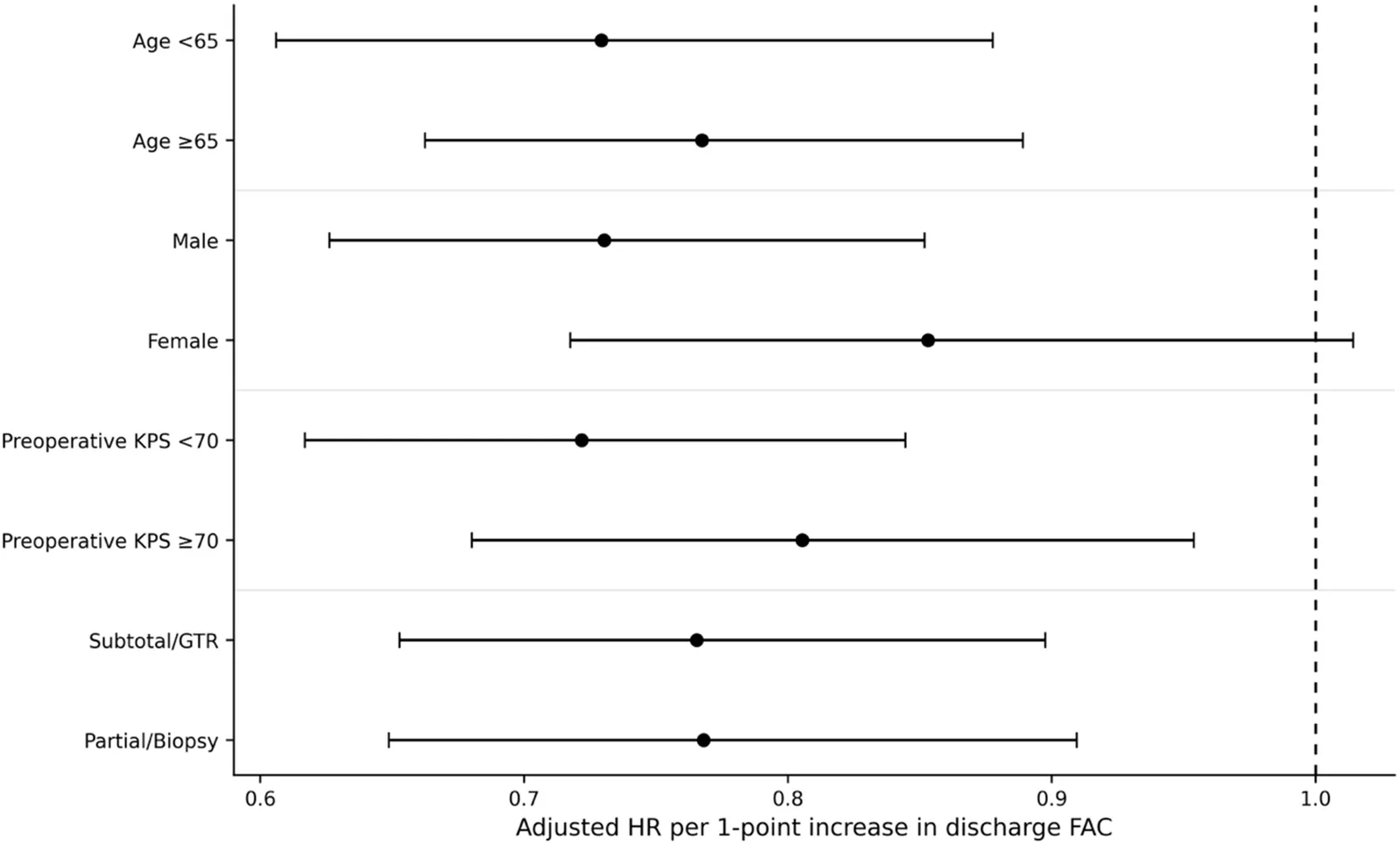
Subgroup analyses of the association between discharge Functional Ambulation Category (FAC) and overall survival. Points indicate adjusted hazard ratios per 1-point increase in discharge FAC, and horizontal bars indicate 95% confidence intervals. Subgroup-specific models were adjusted for the remaining variables in the reference clinical model, excluding the stratification variable itself. The dashed vertical line indicates hazard ratio (HR) = 1. Interaction p values were 0.783 for age, 0.283 for sex, 0.141 for preoperative Karnofsky Performance Status (KPS), and 0.279 for extent of resection. GTR, gross total resection

Secondary subgroup analyses examined age, sex, preoperative KPS, and EOR (Fig. 2). For interaction analyses, discharge FAC was entered per 1-point increase. The inverse association was present in every subgroup (adjusted HRs, 0.72–0.85), with no significant interaction (all *p* ≥ 0.14); the age interaction was *p* = 0.783, arguing against the association being explained solely by the younger age of patients with FAC 5.

In the inter-rater reliability subsample (*n* = 71), linear weighted Cohen’s kappa for discharge FAC was 0.819 (95% CI, 0.735–0.882); full agreement metrics are provided in Online Resource 1.

## Discussion

In this study of 185 patients with newly diagnosed GBM, higher FAC was associated with longer OS at all six peri-treatment assessments, with the strongest association at discharge among in-hospital assessments. The primary discharge-landmark model aligned the survival origin with the FAC assessment; FAC 2–4 and FAC 5 were associated with lower mortality, compared with FAC 0–1, after adjustment for age, sex, EOR, and contemporaneous discharge KPS. Adding FAC improved model fit and modestly improved discrimination, indicating prognostic information beyond global performance status measured at the same time point.

The stronger association at discharge does not indicate that timing itself determines prognosis. Discharge FAC is measured after surgery, early postoperative recovery, rehabilitation, and a substantial portion of the initial CRT course, and is therefore best regarded as an integrated clinical marker at a meaningful transition point, rather than a purely baseline prognostic factor. Systematic landmark analyses supported this interpretation, with the FAC association strengthening from the early postoperative period through discharge.

Discharge KPS is itself prognostic in GBM [25]. In our primary model, discharge KPS and FAC were independently associated with survival, and adding the FAC improved the AIC by 8.4 and C-index by 0.022. Because discrimination remained moderate, the FAC should complement, not replace, global performance status; the two measures provide overlapping but non-identical prognostic information.

Prior studies have yielded mixed findings for walking-related measures. Baseline balance performance was associated with subsequent loss of walking ability and mortality, whereas the 10-m walk test in the same cohort and the 6-min walk test in recurrent malignant glioma cohort were not independent survival predictors [8, 18]. Our findings extend this evidence to an assistance-based, non-timed walking measure.

Discharge FAC may also reflect treatment readiness: patients with higher FAC were more likely to initiate post-discharge adjuvant temozolomide. Adjustment for adjuvant temozolomide did not materially alter FAC estimates; however, the absence of exact initiation dates prevents full separation of functional status from subsequent treatment selection. Thus, the findings establish prognostic association, rather than a causal effect of walking recovery or rehabilitation on survival.

Functional recovery after glioma treatment is multidimensional, and studies have associated inpatient mobility, ADL, discharge-related function, and broader functional outcomes with survival, quality of life, and discharge destination [26–29]. The FAC does not capture all domains but offers a simple bedside measure of walking independence within this broader recovery process. Rehabilitation and supportive-care approaches in glioma remain heterogeneous, and optimal program content, timing, and outcome measures are not yet well established [30–34].

The longitudinal FAC trajectory remains clinically informative. Walking ability was generally lowest immediately after surgery and improved toward discharge and post-discharge follow-up. In our institution, patients commonly remain hospitalized during initial CRT and continuous rehabilitation, which accounts for the long index hospitalization and should be considered when generalizing the timing of discharge assessment. Evidence on continuous rehabilitation through early CRT [11, 12], short-term intensive rehabilitation after postsurgical radiotherapy [35], early mobilization after glioma surgery [36], and rehabilitation outcomes in brain tumor populations [37] supports the clinical relevance of functional recovery during early treatment. Early postoperative worsening should therefore not automatically be interpreted as the patient’s eventual functional level.

Post-discharge FAC at 1 and 4 months was also associated with OS but only among survivors evaluable at those time points. Discharge FAC is available at the transition from inpatient treatment to post-discharge care, before further survivor selection accumulates, supporting its clinical applicability without establishing it as inherently superior to later assessments.

This study has some limitations. First, it was a retrospective, single-center study. GBM was defined histopathologically according to the criteria in use at diagnosis, and the cohort was not systematically reclassified under the 2021 WHO Classification. IDH testing became routine only from 2016 onward, and MGMT promoter methylation was not routinely assessed; therefore, molecular data were unavailable for a substantial, treatment-era-dependent portion of the cohort and could not be included in adjusted analyses. Some historically diagnosed GBMs may be classified differently under contemporary criteria, including astrocytoma, IDH-mutant, WHO grade 4, leaving possible molecular confounding. Second, the FAC was reconstructed retrospectively from clinical documentation, rather than prospectively rated at standardized bedside examinations. The reliability substudy showed good agreement for chart-based assignment but does not establish reliability of prospective direct observation, and misclassification remains possible near adjacent categories. Third, treatment was heterogeneous, including upfront bevacizumab and an age-based change in radiotherapy practice after July 2018. Randomized trials of upfront bevacizumab in newly diagnosed GBM did not show an OS benefit despite improved progression-free survival [38, 39]. FAC estimates were stable after bevacizumab adjustment and restriction to standard CRT without upfront bevacizumab; however, radiotherapy regimen and treatment era were not modeled; residual confounding by treatment selection and era cannot be excluded. Fourth, corticosteroid exposure could not be standardized across FAC assessments because dose, duration, timing, and tapering varied and could not be reconstructed accurately; residual confounding remains possible. Fifth, post-discharge adjuvant temozolomide lacked initiation dates and could not be modeled as a time-dependent exposure; its sensitivity-analysis coefficient is susceptible to immortal-time bias and should not be interpreted as a treatment effect. Sixth, the three-group FAC representation was selected after functional-form assessment and requires external validation. The FAC assesses assistance required for ambulation but not gait speed or endurance, strength, balance, language, or higher cortical function. Future multicenter prospective studies should validate discharge FAC and examine its incremental value with neurological, oncological, and supportive-care factors [32, 37]. Finally, the incremental gain in discrimination was modest, and the findings support prognostic association, rather than a validated clinical prediction model or a causal effect of rehabilitation on survival.

## Conclusion

Among six peri-treatment assessments, discharge FAC showed the strongest association with survival among in-hospital assessments. In a discharge-landmark analysis, it remained associated with post-discharge survival after adjustment for contemporaneous discharge KPS and provided modest incremental prognostic information beyond standard clinical factors. The association was directionally consistent across prespecified subgroups, with no evidence of interaction by age, sex, preoperative KPS, or EOR. The FAC may therefore serve as a practical walking-related marker at the transition to post-discharge care, although causal effects of functional improvement on survival require prospective study.

## Supplementary Information

Below is the link to the electronic supplementary material.

Supplementary Material 1

Supplementary Material 2

## Data Availability

No datasets were generated or analysed during the current study.

## Code availability

The analysis code is available from the corresponding author upon reasonable request, subject to institutional policies.

## Declarations

### Patient and public involvement

Patients and the public were not involved in the design, conduct, reporting, or dissemination plans of this retrospective study.

## References

[1] Wen PY, Weller M, Lee EQ et al (2025) Glioblastoma in adults: A Society for Neuro-Oncology (SNO) and European Society of Neuro-Oncology (EANO) consensus review on current management and future directions. Neuro Oncol 27:2751–2788. 10.1093/neuonc/noaf177 PMC12908567 40827022

[2] Schaff LR, Mellinghoff IK (2023) Glioblastoma and other primary brain malignancies in adults: A review. JAMA 329:574–587. 10.1001/jama.2023.0023 PMC11445779 36809318

[3] Stupp R, Mason WP, van den Bent MJ et al (2005) Radiotherapy plus concomitant and adjuvant temozolomide for glioblastoma. N Engl J Med 352:987–996. 10.1056/NEJMoa043330 15758009

[4] Conrad K, Löber-Handwerker R, Hazaymeh M, Rohde V, Malinova V (2024) Personalized prognosis stratification of newly diagnosed glioblastoma applying a statistical decision tree model. J Neurooncol 168:425–433. 10.1007/s11060-024-04683-6 PMC11186892 38639854

[5] Hegi ME, Diserens AC, Gorlia T et al (2005) MGMT gene silencing and benefit from temozolomide in glioblastoma. N Engl J Med 352:997–1003. 10.1056/NEJMoa043331 15758010

[6] Gorlia T, van den Bent MJ, Hegi ME et al (2008) Nomograms for predicting survival of patients with newly diagnosed glioblastoma: prognostic factor analysis of EORTC and NCIC trial 26981–22981/CE.3. Lancet Oncol 9:29–38. 10.1016/S1470-2045(07)70384-4 18082451

[7] Gállego Pérez-Larraya JG, Ducray F (2014) Treating glioblastoma patients with poor performance status: where do we go from here? CNS Oncol 3:231–241. 10.2217/cns.14.20 PMC6124361 25055131

[8] Ruden E, Reardon DA, Coan AD et al (2011) Exercise behavior, functional capacity, and survival in adults with malignant recurrent glioma. J Clin Oncol 29:2918–2923. 10.1200/JCO.2011.34.9852 PMC3138718 21690470

[9] Roberts PS, Nuño M, Sherman D et al (2014) The impact of inpatient rehabilitation on function and survival of newly diagnosed patients with glioblastoma. PM R 6:514–521. 10.1016/j.pmrj.2013.12.007 24384359

[10] Tang V, Rathbone M, Park Dorsay J, Jiang S, Harvey D (2008) Rehabilitation in primary and metastatic brain tumours: impact of functional outcomes on survival. J Neurol 255:820–827. 10.1007/s00415-008-0695-z 18500499

[11] Natsume K, Sakakima H, Kawamura K et al (2022) Factors influencing the improvement of activities of daily living during inpatient rehabilitation in newly diagnosed patients with glioblastoma multiforme. J Clin Med 11:417. 10.3390/jcm11020417 PMC8780839 35054111

[12] Natsume K, Yoshida A, Sakakima H et al (2024) Age-independent benefits of postoperative rehabilitation during chemoradiotherapy on functional outcomes and survival in patients with glioblastoma. J Neurooncol 170:129–137. 10.1007/s11060-024-04785-1 PMC11447139 39078543

[13] Vargo M (2011) Brain tumor rehabilitation. Am J Phys Med Rehabil 90:S50–S62. 10.1097/PHM.0b013e31820be31f 21765264

[14] Thakkar P, Greenwald BD, Patel P (2020) Rehabilitation of adult patients with primary brain tumors: a narrative review. Brain Sci 10:492. 10.3390/brainsci10080492 PMC7464729 32751074

[15] Holden MK, Gill KM, Magliozzi MR, Nathan J, Piehl-Baker L (1984) Clinical gait assessment in the neurologically impaired. Reliab meaningfulness Phys Ther 64:35–40. 10.1093/ptj/64.1.35 6691052

[16] Mehrholz J, Wagner K, Rutte K, Meissner D, Pohl M (2007) Predictive validity and responsiveness of the Functional Ambulation Category in hemiparetic patients after stroke. Arch Phys Med Rehabil 88:1314–1319. 10.1016/j.apmr.2007.06.764 17908575

[17] Ishida N, Nikaido Y, Urakami H et al (2026) Early postoperative predictors of independent walking in individuals with brain tumors. Arch Phys Med Rehabil 107:988–996. 10.1016/j.apmr.2025.09.036 41161465

[18] Liljehult MM, Buus L, Liljehult J, Rasmussen BK (2017) Walking ability in patients with glioblastoma: prognostic value of the Berg Balance Scale and the 10 meter walk test. J Neurooncol 135:335–342. 10.1007/s11060-017-2579-5 28752499

[19] Rakovec M, Myneni S, Johnson S et al (2024) Activity Measure for Post-Acute care (AM-PAC) scores predict Short and Long-Term outcomes following glioblastoma resection. J Clin Neurosci 127:110746. 10.1016/j.jocn.2024.07.007 39079422

[20] Louis DN, Perry A, Wesseling P et al (2021) The 2021 WHO Classification of Tumors of the Central Nervous System: a summary. Neuro Oncol 23:1231–1251. 10.1093/neuonc/noab106 PMC8328013 34185076

[21] Bartolo M, Zucchella C, Pace A et al (2012) Early rehabilitation after surgery improves functional outcome in inpatients with brain tumours. J Neurooncol 107:537–544. 10.1007/s11060-011-0772-5 22124725

[22] Harrell FE Jr, Lee KL, Mark DB (1996) Multivariable prognostic models: issues in developing models, evaluating assumptions and adequacy, and measuring and reducing errors. Stat Med 15:361–387. 10.1002/(SICI)1097-0258(19960229)15:4<361::AID-SIM168>3.0.CO;2-4 8668867

[23] Akaike H (1974) A new look at the statistical model identification. IEEE Trans Autom Control 19:716–723. 10.1109/TAC.1974.1100705

[24] von Elm E, Altman DG, Egger M et al (2007) The Strengthening the Reporting of Observational Studies in Epidemiology (STROBE) statement: guidelines for reporting observational studies. Ann Intern Med 147:573–577. 10.7326/0003-4819-147-8-200710160-00010 17938396

[25] Sasaki S, Tsukamoto S, Ishida Y et al (2024) The Karnofsky Performance Status at discharge is a prognostic indicator of life expectancy in patients with glioblastoma. Cureus 16:e66226. 10.7759/cureus.66226 PMC11376000 39238708

[26] Gruchala T, Lewis CW, Abplanalp K et al (2025) Predicting medical prognosis in patients with glioblastoma during inpatient rehabilitation using bed mobility function. PM R 17:1343–1353. 10.1002/pmrj.13402 PMC12354043 40386896

[27] Watanabe T, Noto S, Natsumeda M et al (2022) Characteristics of health-related quality of life and related factors in patients with brain tumors treated with rehabilitation therapy. J Patient Rep Outcomes 6:94. 10.1186/s41687-022-00499-y PMC9448840 36068453

[28] Shibahashi H, Murakawa M, Matsuda K, Takakubo Y, Takagi M (2024) Barthel index and age as predictors of discharge destination in patients with glioblastoma. Cancer Investig 42:619–626. 10.1080/07357907.2024.2371367 38934568

[29] De Witt Hamer PC, Klein M, Hervey-Jumper SL, Wefel JS, Berger MS (2021) Functional outcomes and health-related quality of life following glioma surgery. Neurosurgery 88:720–732. 10.1093/neuros/nyaa365 PMC7955971 33517431

[30] Vargo M, Henriksson R, Salander P (2016) Rehabilitation of patients with glioma. Handb Clin Neurol 134:287–304. 10.1016/B978-0-12-802997-8.00017-7 26948361

[31] Crooms RC, Goldstein NE, Diamond EL, Vickrey BG (2020) Palliative care in high-grade glioma: a review. Brain Sci 10:723. 10.3390/brainsci10100723 PMC7599762 33066030

[32] Spina S, Facciorusso S, Cinone N, Pellegrino R, Fiore P, Santamato A (2023) Rehabilitation interventions for glioma patients: a mini-review. Front Surg 10:1137516. 10.3389/fsurg.2023.1137516 PMC10313351 37396290

[33] Pieczyńska A, Zasadzka E, Pilarska A, Procyk D, Adamska K, Hojan K (2023) Rehabilitation exercises supported by monitor-augmented reality for patients with high-grade glioma undergoing radiotherapy: results of a randomized clinical trial. J Clin Med 12:6838. 10.3390/jcm12216838 PMC10648373 37959303

[34] Zanotto A, Glover RN, Zanotto T, Boele FW (2024) Rehabilitation in people living with glioblastoma: a narrative review of the literature. Cancers (Basel) 16:1699. 10.3390/cancers16091699 PMC11083409 38730651

[35] Matsutani M, Ideguchi M, Kitamura T et al (2021) Short-term intensive rehabilitation following postsurgical radiotherapy for patients with newly diagnosed glioblastoma associated with physical disabilities. Jpn J Neurosurg 30:859–870. 10.7887/jcns.30.859

[36] Chen X, Wan L, Wang B (2025) Early mobilization in postoperative glioma patients: real-world impact on recovery and long-term prognosis. Sci Rep 15:18032. 10.1038/s41598-025-01871-w PMC12102244 40410289

[37] Pieczyńska A, Pilarska A, Hojan K (2022) Predictors of functional outcomes in adults with brain tumor undergoing rehabilitation treatment: a systematic review. Eur J Phys Rehabil Med 58:666–674. 10.23736/S1973-9087.22.07510-4 PMC10019483 35801976

[38] Gilbert MR, Dignam JJ, Armstrong TS et al (2014) A randomized trial of bevacizumab for newly diagnosed glioblastoma. N Engl J Med 370(8):699–708. 10.1056/NEJMoa1308573 PMC4201043 24552317

[39] Chinot OL, Wick W, Mason W et al (2014) Bevacizumab plus radiotherapy–temozolomide for newly diagnosed glioblastoma. N Engl J Med 370:709–722. 10.1056/NEJMoa1308345 24552318

