# Supplementary material for: Prognostic significance of walking ability at hospital discharge for overall survival in newly diagnosed glioblastoma: a comparison across six peri-treatment assessments
Source: J Neurooncol. 2026 Sep 11;179(2):78. doi: 10.1007/s11060-026-05784-0 (PMC13569522; doi:10.1007/s11060-026-05784-0)
Supplement: Supplementary file 1 [file 11060_2026_5784_MOESM1_ESM.pdf]

Online Resource 1

Article title: Prognostic Significance of Walking Ability at Hospital Discharge for Overall Survival in Newly Diagnosed Glioblastoma: A Comparison Across Six Peri-treatment Assessments

Journal name: *Journal of Neuro-Oncology*

Shuhei Tokiwa[1, 2†], Keisuke Natsume[1†], Akira Yoshida[1, 2*], Hajime Yonezawa[3], Kentaro Kawamura[1, 2], Kazuki Nakanishi[4], Nayuta Higa[3], Ryosuke Hanaya[3], and Megumi Shimodozono[1, 2]

[1]Department of Rehabilitation, Kagoshima University Hospital, Kagoshima, Japan

[2]Department of Rehabilitation and Physical Medicine, Kagoshima University Graduate School of Medical and Dental Sciences, Kagoshima, Japan

[3]Department of Neurosurgery, Kagoshima University Graduate School of Medical and Dental Sciences, Kagoshima, Japan

[4]Department of Physical Therapy, School of Health Sciences, Faculty of Medicine, Kagoshima University, Kagoshima, Japan

[†]These authors contributed equally to this work.

***Corresponding author:** Akira Yoshida, MD, PhD

Department of Rehabilitation and Physical Medicine, Kagoshima University Graduate School of Medical and Dental Sciences, 8–35–1, Sakuragaoka, Kagoshima 890–8520, Japan

Phone: +81–99–275–5339

Fax: +81–99–275–1273

E-mail: akiray@m.kufm.kagoshima-u.ac.jp

Supplementary Methods

Study cohort and exclusion criteria

Of 225 screened admissions, 40 cases outside the predefined index-care pathway were excluded: non-GBM final diagnosis after biopsy (n=4), postoperative admissions outside the pathway (n=2), surgery alone (n=7), surgery with chemotherapy alone (n=5), surgery with radiotherapy alone (n=8), chemotherapy alone (n=5), bevacizumab-containing non-standard regimens without standard CRT (n=3), no surgery or tumor-directed treatment (n=2), no inpatient rehabilitation (n=1), and other clinical trial participation (n=3). The final analytic cohort comprised 185 patients.

Analysis framework

The Functional Ambulation Category (FAC) and retrospective assignment: The FAC is a six-point ordinal scale graded by the degree of human assistance required: 0, nonfunctional ambulation; 1, continuous manual support; 2, continuous or intermittent light touch; 3, supervision or verbal cueing without physical contact; 4, independent walking on level surfaces only; and 5, independent walking on all surfaces. Higher scores indicate greater walking independence. FAC was assessed preoperatively, on postoperative day 5 (±1), before chemoradiotherapy (CRT), at hospital discharge, and 1 and 4 months after discharge. For the main study, each record was retrospectively scored once by one physical therapist using rehabilitation records, physicians' notes, and nursing documentation of daily-life mobility.

Screening analyses used survival from surgery. The primary discharge-landmark model used survival from discharge and complete cases for discharge FAC, age, sex, extent of resection (EOR), and discharge Karnofsky Performance Status (KPS) (n=162; 138 deaths). Missing data were not imputed.

Functional form of discharge FAC

Discharge FAC was compared as a linear term, six-level categorical variable, three-group classification (0–1, 2–4, and 5), and clinically relevant dichotomizations in the same adjusted model using Akaike information criterion, Harrell's C-index, and likelihood-ratio tests. The three groups represented nonfunctional or severely dependent ambulation (0–1), intermediate assisted or limited independent ambulation (2–4), and independent ambulation on all surfaces (5).

Systematic landmark analyses

Landmark Cox models used surgery, postoperative day 5, CRT initiation, and hospital discharge as the respective time origins for the four in-hospital FAC assessments, with adjustment for age, sex, preoperative KPS, and EOR. For the common-basis comparison of the four in-hospital assessments, each three-group FAC measure was added separately to a fixed clinical reference model comprising age, sex,

preoperative KPS, and EOR, within a common complete-case subset (n=146; 128 deaths). Post-discharge FAC assessments were treated as exploratory because they required survival and evaluability at later time points. FAC was grouped as 0–1, 2–4, and 5; FAC 5 was not observed on postoperative day 5.

Sensitivity analyses

Sensitivity analyses additionally adjusted for upfront bevacizumab, restricted the cohort to standard CRT without upfront bevacizumab, modeled EOR categorically, stratified the Cox model by EOR when addressing non-proportionality, and adjusted for post-discharge adjuvant temozolomide (TMZ). Because exact TMZ initiation dates were unavailable, TMZ was modeled as a fixed covariate for sensitivity analysis only.

Proportional hazards assessment

The proportional hazards assumption was assessed using scaled Schoenfeld residuals. The assumption was satisfied for discharge FAC (p=0.129) but not for EOR (p=0.006). Therefore, a sensitivity Cox model was fitted with EOR entered as a stratification factor, rather than as a covariate.

Inter-rater reliability substudy

Paired discharge FAC ratings were available for 71 patients in a convenience subsample. The main-study rating had been assigned by one physical therapist. A second rating had been assigned separately by another physical therapist for an unrelated retrospective investigation; the second rater did not participate in the present survival analysis, did not refer to the main-study rating, and was unaware of survival outcomes. Thus, each record was scored once within each dataset and the two ratings were independently generated. Agreement was assessed using linear weighted Cohen's kappa as the primary statistic because FAC is ordinal, with a two-way random-effects, absolute-agreement, single-measure intraclass correlation coefficient [ICC(2,1)] as a supplementary metric. Ninety-five percent confidence intervals were obtained by patient-level nonparametric bootstrap resampling (5,000 repetitions). Agreement of the three-group FAC classification (0–1/2–4/5) was also evaluated.

Supplementary Table S1. Functional-form comparison of discharge FAC

FAC representation	FAC parameters added	AIC	C-index	LR *p* vs. clinical model
Clinical model without FAC	0	1134.77	0.692	Reference
Linear FAC, per 1-point increase	1	1131.89	0.705	0.027
Six-level categorical FAC (0 reference)	5	1125.03	0.718	0.001
Three groups: 0–1 / 2–4 / 5	2	1126.37	0.715	0.002
FAC ≥1	1	1133.32	0.693	0.063
FAC ≥2	1	1125.98	0.702	0.001
FAC ≥3	1	1134.64	0.696	0.144
FAC ≥4	1	1136.63	0.689	0.710
FAC ≥5	1	1134.47	0.704	0.129

All models used the same 162 complete cases (138 deaths) and adjusted for age, sex, EOR, and discharge KPS. The six-level model fit better than the linear model (LR p=0.00499), whereas fit did not differ significantly between the six-level and three-group models (LR p=0.0616).

AIC, Akaike information criterion; EOR, extent of resection; FAC, Functional Ambulation Category; KPS, Karnofsky Performance Status; LR, likelihood ratio

Supplementary Table S2. Systematic landmark analyses of in-hospital FAC assessments

FAC assessment/landmark	n/deaths	FAC 2–4 vs. 0–1, adjusted HR (95% CI)	FAC 5 vs. 0–1, adjusted HR (95% CI)	LR *p* for FAC	ΔAIC	ΔC-index
Preoperative/surgery	147/129	0.830 (0.514–1.341)	0.782 (0.364–1.681)	0.740	+3.40	+0.006
Postoperative day 5	150/131	0.683 (0.458–1.018)	Not observed	0.060	−1.55	−0.002
Before CRT	150/131	0.580 (0.377–0.891)	0.452 (0.235–0.869)	0.025	−3.40	+0.013
Discharge	149/130	0.364 (0.232–0.572)	0.255 (0.147–0.443)	<0.001	−22.05	+0.045

Models adjusted for age, sex, preoperative KPS, and ordinal EOR. On postoperative day 5, FAC 5 was not observed. Negative ΔAIC indicates improved fit versus the corresponding clinical model without FAC.

AIC, Akaike information criterion; CI, confidence interval; CRT, chemoradiotherapy; EOR, extent of resection; FAC, Functional Ambulation Category; HR, hazard ratio; KPS, Karnofsky Performance Status; LR, likelihood ratio

Supplementary Table S3. Sensitivity analyses of the discharge-landmark model

Sensitivity model	n/deaths	FAC 2–4 vs. 0–1, adjusted HR (95% CI)	FAC 5 vs. 0–1, adjusted HR (95% CI)	Additional estimate/note	C-index
Primary model	162/138	0.445 (0.271–0.730)	0.327 (0.169–0.632)	Reference	0.715
+upfront bevacizumab	162/138	0.441 (0.266–0.730)	0.326 (0.169–0.630)	Bevacizumab: HR 0.956 (0.602–1.517), p=0.848	0.716
Standard CRT only (no upfront bevacizumab)	108/94	0.424 (0.234–0.768)	0.333 (0.152–0.729)	Restricted cohort	0.723
+post-discharge adjuvant TMZ	148/127	0.438 (0.255–0.752)	0.330 (0.162–0.674)	Adjuvant TMZ: HR 0.423 (0.249–0.717), p=0.001	0.711
EOR modeled categorically	162/138	0.464 (0.278–0.775)	0.340 (0.174–0.667)	Biopsy reference for EOR categories	0.717
EOR as stratification factor	162/138	0.487 (0.289–0.820)	0.364 (0.181–0.730)	Stratified by EOR	—

The primary model adjusted for age, sex, ordinal EOR, and discharge KPS. In the EOR-stratified sensitivity model, EOR was used as the stratification factor rather than entered as a covariate; its C-index is not shown because the baseline hazard was allowed to differ across EOR strata. Adjuvant TMZ was modeled as a fixed covariate because exact initiation dates were unavailable.

CRT, chemoradiotherapy; CI, confidence interval; EOR, extent of resection; FAC, Functional Ambulation Category; HR, hazard ratio; KPS, Karnofsky Performance Status; TMZ, temozolomide

Supplementary Table S4. Inter-rater reliability of retrospectively assigned discharge FAC

Metric	Result
Paired ratings	71
Exact agreement	67.6%
Agreement within ±1 FAC level	94.4%
Linear weighted Cohen kappa	0.819 (95% CI, 0.735–0.882)
ICC(2,1)	0.933 (95% CI, 0.888–0.962)
Three-group exact agreement	80.3%
Three-group linear weighted kappa	0.764 (95% CI, 0.636–0.867)

This substudy assessed chart-based retrospective FAC assignment.

CI, confidence interval; FAC, Functional Ambulation Category; ICC(2,1), two-way random-effects, absolute-agreement, single-measure intraclass correlation coefficient.
